# Fast quantitative determination of microbial rhamnolipids from cultivation broths by ATR-FTIR Spectroscopy

**DOI:** 10.1186/1754-1611-2-13

**Published:** 2008-10-07

**Authors:** Frank Leitermann, Christoph Syldatk, Rudolf Hausmann

**Affiliations:** 1 Research University Karlsruhe, Institute of Engineering in Life Sciences, Section of Technical Biology, Engler-Bunte-Ring 1, 76131 Karlsruhe, Germany

## Abstract

**Background:**

Vibrational spectroscopic techniques are becoming increasingly important and popular because they have the potential to provide rapid and convenient solutions to routine analytical problems. Using these techniques, a variety of substances can be characterized, identified and also quantified rapidly.

**Results:**

The rapid ATR-FTIR (Attenuated Total Reflectance Fourier Transform Infrared Spectroscopy) *in time *technique has been applied, which is suitable to quantify the concentrations of microbial rhamnolipids in a typical cultivation process. While the usually applied HPLC analysis requires an extensive and time consuming multi step extraction protocol for sample preparation, the ATR-FTIR-method allows the quantification of the rhamnolipids within 20 minutes. Accuracies between 0.5 g/l – 2.1 g/l for the different analytes were determined by cross validation of the calibration set. Even better accuracies between 0.28 g/l – 0.59 g/l were found for independent test samples of an arbitrarily selected cultivation.

**Conclusion:**

ATR-FTIR was found to be suitable for the rapid analysis of rhamnolipids in a biotechnological process with good reproducibility in sample determination and sufficient accuracy. An improvement in accuracy through continuous expansion and validation of the reference spectra set seems very likely.

## Background

Ranging from bulk chemicals like ethanol to high value proteins, biotechnological production processes are an increasingly important manufacturing route for various products [1]. The control of these bioprocesses often can be considered as suboptimal. Usually only a few parameters, like pH, pO_2 _and temperature, are monitored online. All additional information required must be gained through analysis of individual samples. Typical assays often rely on enzymatic reactions or separation techniques such as high performance liquid chromatography (HPLC) [2]. Therefore, the analysis results often will be available only with a significant time delay.

Hence, vibrational spectroscopic techniques that provide rapid and convenient solutions to routine analytical problems are being increasingly adopted. A variety of substances can be characterized, identified and also quantified rapidly in parallel from a single sample spectrum [3]. Fourier transform infrared spectroscopy (FTIR) is a reliable and well-recognized method [4]. For a complex analyte-matrix combination, like that which occurs in fermentations broths, the adaptation of the FTIR technique needs extensive experience and time. Nevertheless, once a method has been established, it allows for relatively fast assays of compounds, where the alternative quantitative analysis (e.g. HPLC methods) can be time-consuming. Attenuated total reflection infrared Fourier transform spectroscopy (ATR-FTIR) is a FTIR variety, which allows organic substances in aqueous solutions to be determined[5]. ATR-FTIR involves the collection of radiation reflected from the interfacial surface between the aqueous solution and a reflection element (ATR crystal). In this crystal, evanescent waves emanate from the crystal, penetrate the aqueous solution and are absorbed by substances in this solution [6,7]. Concerning the analysis of biosurfactants several spectroscopic methods for the characterization, identification and quantification have been reported [8-13].

Biosurfactants are microbially produced surface active compounds [14]. Anionic glycolipid biosurfactants consisting of L-rhamnose sugars and aliphatic chains moieties are called rhamnolipids. Several variations of these rhamnolipids are known [15]. Rhamnolipids produced by *P. aeruginosa *strains are often composed of one or two L-rhamnose and, additionally, one or two β-hydroxydecanoic acid moieties. They are termed rhamnolipid 1 (L-rhamnosyl-3-hydroxydecanoyl-3-hydroxydecanoate) and rhamnolipid 3 (L-rhamnosylrhamnosyl-3-hydroxydecanoyl-3-hydroxydecanoate) [16].

The objective of the presented work is to highlight the range of application made possible by utilizing an ATR-FTIR method for the monitoring of a biotechnological process for the production of microbial rhamnolipids. Additionally, the potential of this method for achieving structural information and identification purposes concerning rhamnolipids was investigated.

## Results

### Characterization and interpretation of the absorbance spectrum of rhamnolipid 3

**Figure 1:**
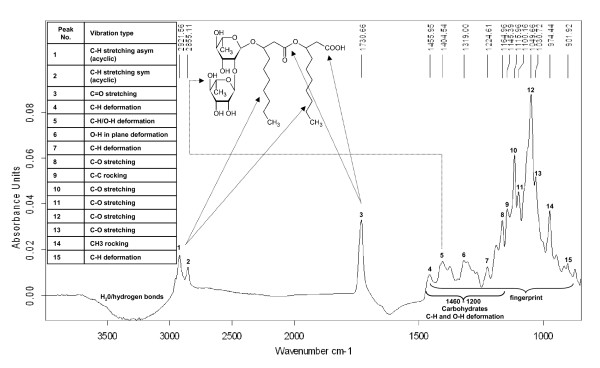
**Rhamnolipid 3 IR-spectrum**. ATR-FTIR spectrum of an aqueous solution (89 mg/mL) of rhamnolipid 3. The allocation of conspicuous absorption to corresponding characteristic group absorptions from literature was attempted.

An ATR-FTIR spectrum of pure rhamnolipid 3 in water was recorded, as shown in Figure 1. For a better understanding of the IR spectrum, subsequently the dominant absorbance bands were correlated to the according group absorbance frequencies. The broad negative bands at about 3300 cm^-1 ^and 1640 cm^-1 ^result from a higher water concentration in the reference spectrum (pure water) relative to the aqueous sample, and are attributed to hydrogen bonding and O-H stretching of water. The double bands at 2921 and 2855 cm^-1 ^are derived from symmetric C-H stretching vibrations of aliphatic groups, like those represented in the hydroxydecanoic acid chain tails of rhamnolipid 3. A C=O stretching band at 1730 cm^-1 ^is characteristic of ester bonds and carboxylic acid groups. In the fingerprint region of the spectrum, the area between 1200 – 1460 cm^-1 ^represents C-H and O-H deformation vibrations, typical for carbohydrates as in the rhamnose units of the molecule, for example. The lower range of the fingerprint region below 1200 cm^-1 ^represents different kinds of C-H, C-O and CH_3 _vibrations which cannot be allocated more closely [6].

### Basic investigations for the applicability of ATR-FTIR

Rhamnolipids are surface active compounds, and, therefore, absorbance effects at the interfacial surface of the ATR crystal to liquid phase must be expected. With respect to the small volume of the sample, 20 μl, even evaporation may affect the absorbance intensity of the spectra and influence the quantification of the analytes. Hence, time dependant spectra of the samples were recorded for comparison. Four spectra of the same sample were recorded in time intervals of 2 minutes. Neither intensity changes of single bands nor an overall intensity change of the absorbance spectrum were observed. Therefore evaporation and interfacial absorbance effects were not relevant for all practical purposes.

**Figure 2:**
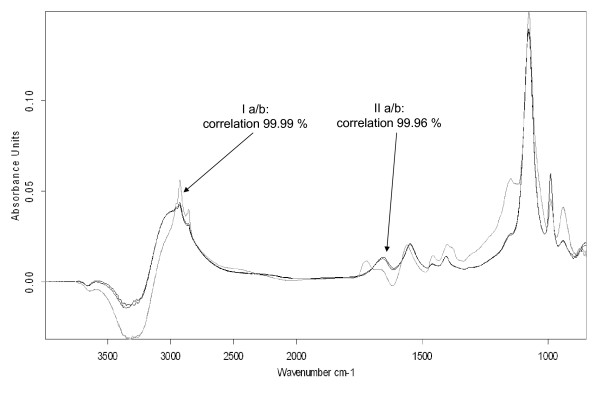
**Reproducibility of spectra measurements**. This figure displays four spectra of two fermentation derived samples after double application. A comparison of the corresponding two spectra of each sample showed correlations of above 99.9%.

For verification of the reproducibility of the application method, two samples of the cultivation broth were prepared and measured twice. The corresponding spectra are shown in Figure 2. The comparison of the corresponding spectra only showed minimal diversities, relating to the spectra intensities., A spectral match was found to be above 99.99%, for the first sample and 99.96%, for the second sample.

### Quantification of rhamnolipids by ATR-FTIR

**Figure 3:**
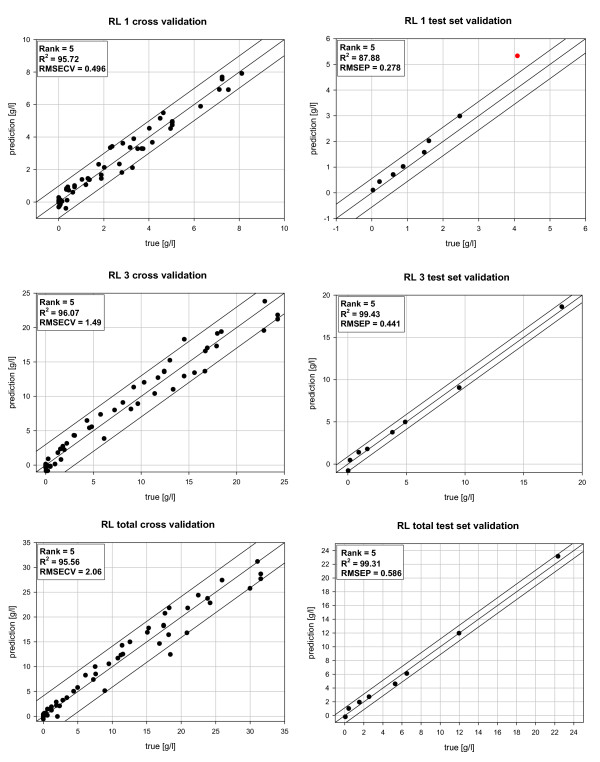
**Validation of the developed PLS based quantification procedure of rhamnolipids**. Results for the predictive quality of the performed cross validations and test set validation for rhamnolipid 1 (RL 1), rhamnolipid 3 (RL 3) and rhamnolipid total (RL total).

To establish multivariate calibration methods, a spectra set of about 80 samples, derived from 9 different rhamnolipid fermentations, was recorded. Using this spectra set, as a first step, three independent calibrations for rhamnolipid 1 and rhamnolipid 3, as well as the combined rhamnolipid 1 and 3 content, were set up. All calibrations were evaluated and optimized by cross validation, according to their determination coefficients R^2^, RMSECV (root mean square error of cross validation) and utilized PLS factors (synonym to the terminus rank). The results for the reference versus the predicted values are displayed in Figure 3. By an applied rank of five, similar determination coefficients R^2 ^from 95.56 to 96.07 were calculated for all three analytes. For rhamnolipid 1 and rhamnolipid 3 a RMSECV of 0.496 g/l respectively 1.49 g/l was determined. For the total rhamnolipid 1 and 3 in the samples, an error of 2.06 g/l was found. For the calibration purposes of all three analytes, a single frequency area from 900 – 1200 cm^-1 ^was selected. As mathematical pre-treatment for rhamnolipid 1 and rhamnolipid 3, the first derivative with the subtraction of a straight line and for rhamnolipid total the first derivative results in the highest determination coefficients and lowest prediction errors.

In a second step, the evaluation of the prediction error of the established calibration models versus an independent test samples set from an arbitrarily selected fermentation process was investigated by test set validations. The results are displayed in Figure 3. At a rank of 5, for rhamnolipid 1, a determination coefficient R^2 ^of 87.88 and a RMSEP of 0.278 g/l were calculated. When considering a rank of 5, the optimized correlation for rhamnolipid 3 responds with a R^2 ^of 99.43 and a RMSEP of 0.441 g/l. For the total rhamnolipid content a R^2 ^of 99.31 and a RMSEP of 0.586, at a rank of 5, were found. Similar to the cross validated models, the best results were found by restricting of a single frequency area from 900 – 1200 cm^-1^. Also, the similar mathematical pre-treatments, as described above, were responding the best results.

## Discussion

The spectrum of pure rhamnolipid 3, as shown in Figure 1, indicates characteristic absorption bands at 2921 and 2855 cm^-1 ^derived from symmetric C-H and a C=O stretching band at 1730 cm^-1^. Gartshore et al. [8] suggested the correlation of a single peak area when analyzing artificially prepared samples of three biosurfactants, lecithin, cholesterol laurate, and spiculi sporic acid. The peak utilized in this study was between 1720 – 1770 cm^-1^. Unlike these spectroscopic experiments, the calibrations derived from the cultivation samples showed the best correlations for the quantitative determination of rhamnolipids when the fingerprint regions of the spectra were utilized. This could be explained by the complexity of the analyte-matrix of real cultivation samples. Besides rhamnolipids, a great variety of additional substances, like plant oil, fatty acids, buffer salts or ester compounds were present, which influence the absorption intensities in the spectrum area of the three characteristic bands upon 1700 cm^-1^. Hence, the unapparent information comprised in the fingerprint area of the spectra was found to be most suitable for selective, quantitative determinations of rhamnolipids.

Overall, the ATR-FTIR assays showed a good reproducibility. Even suspected interfacial or evaporation effects could not be observed, as can be seen from the consecutive spectra shown in Figures 2 and 3. The differentiation of rhamnolipid 1, rhamnolipid 3 and the total rhamnolipid was possible.

By cross validation, predictive errors between 0.496 g/l to 2.06 g/l were determined, which are adequate for a fast *in time *analysis. Similar or even better results, with predictive errors from 0.278 g/l to 0.586 g/l, were obtained by test set validation of independent cultivation samples. Accounting for the highest rhamnolipid 1 value in Figure 3, resulted from a possible outlier, the derived correlation error seems to be even lower. Also, it has been shown that reliable correlations could be found, despite different fermentation conditions with respect to the substrates. An improvement of the accuracy and stability of the correlations by expansion of the calibrations data sets with samples from additional cultivations is expected. In addition, the major advantage of an ATR-FTIR compared to a HPLC quantification of rhamnolipids is the time needed. Whereas HPLC data is available after approximately 24 h, caused by multiple extractions, evaporation and derivatisation steps, ATR-FTIR provides process values within 20 minutes.

## Conclusion

ATR-FTIR was found to be appropriate for the rapid analysis of rhamnolipids in a biotechnological process with sufficient accuracy and good reproducibility. Further improvements to the quantification accuracy can be expected by expansion of the calibration data set with samples from additional cultivations. By opening a "spectroscopic eye" into bioprocesses for the production of microbial rhamnolipids, *in time *ATR-FTIR monitoring makes rapid process characterization and process control much easier. Future objectives for the modifications of the utilized ATR-FTIR technique towards an online *in situ *measurement system by using integrated ATR probes seemsto be very promising.

## Methods

### Microorganism

*Pseudomonas aeruginosa *DSM 7108 was obtained from the German Collection of Microorganisms and Cell Cultures. The strain is stored as glycerol stock culture at -80°C.

### Media

For seed cultures, a lysogeny broth (LB) medium containing 5 g/l NaCl was used [17]. For the bioreactor cultivations, a Ca-free mineral salt medium was used. The medium is based on a 0.1 M sodium phosphate buffer pH 6.5 supplemented with 0.5 g/l MgSO_4 _× 7 H_2_O, 1 g/l KCl and 15 g/l NaNO_3 _(modified, according to [18]). The phosphate buffer was separately autoclaved from the other salts. A sterile filtered trace elements solution consisted of 2 g/l sodium citrate × 2 H_2_O, 0.28 g/l FeCl_3 _× 6 H_2_O, 1.4 g/l ZnSO_4 _× 7 H_2_O, 1.2 g/l CoCl_2 _× 6H_2_O, 1.2 g/l CuSO_4 _× 5 H_2_O and 0.8 g/l MnSO_4 _× H_2_O was added to the media as described below. All chemicals were of analytical grade. As a carbon source, three types of sunflower oils (high oleic sunflower oils HOS 90+, HOS 80+, conventional sunflower oil), linseed oil, rapeseed oil, corn oil soy bean oil (all by courtesy of Dr. B Schlüter, Unternehmensberatung und Dienstleistung im vor- und nachgelagerten Bereich der Landwirtschaft, Bornheim, Germany) and fish oil (by courtesy of Dr. Krumbholz, KD-Pharma Bexbach GmbH, Bexbach, Germany) of technical/food grade were used.

### Cultivation conditions

As for the bioreactor cultures, 25 ml LB medium was inoculated with 100 μl of a glycerol stock culture in a 100 ml shaking flask. This culture was incubated for 12 h at 30°C and 140 rpm in an incubation shaker (HT Infors Multitron II), until an optical density at 580 nm (OD_580_) of 2.5 – 3.0 was reached (Amersham Biosciences Ultrospec 1100pro). With 2 ml of this seed culture, the main cultures were inoculated. The cultivations were performed in a 500 ml parallel bioreactor system (Sixfors, IFORS GmbH Einsbach, Germany) with 214 ml (200 ml media + 14 ml plant oil) initial volume. The temperature was controlled and maintained at 30°C. During the process, the pH was controlled and maintained at 6.5. An additional 14 ml substrate was added after approximately 40 h of process time. One ml per liter of the trace element solution was added at distinct process times (t = 0; 20; 40; 70; 120 h). The reactors were equipped with additional 4 blade Rushton stirrers with blade geometry of 1:1 for mechanical foam destruction. For chemical defoaming Contraspum™ A4050 (Zschimmer&Schwarz GmbH & Co KG Chemische Fabriken, Lahnstein, Germany) was used. The processes were controlled and recorded by a personal computer system with the process software Iris (INFORS GmbH Einsbach, Germany).

### Sampling protocol

**Figure 4:**
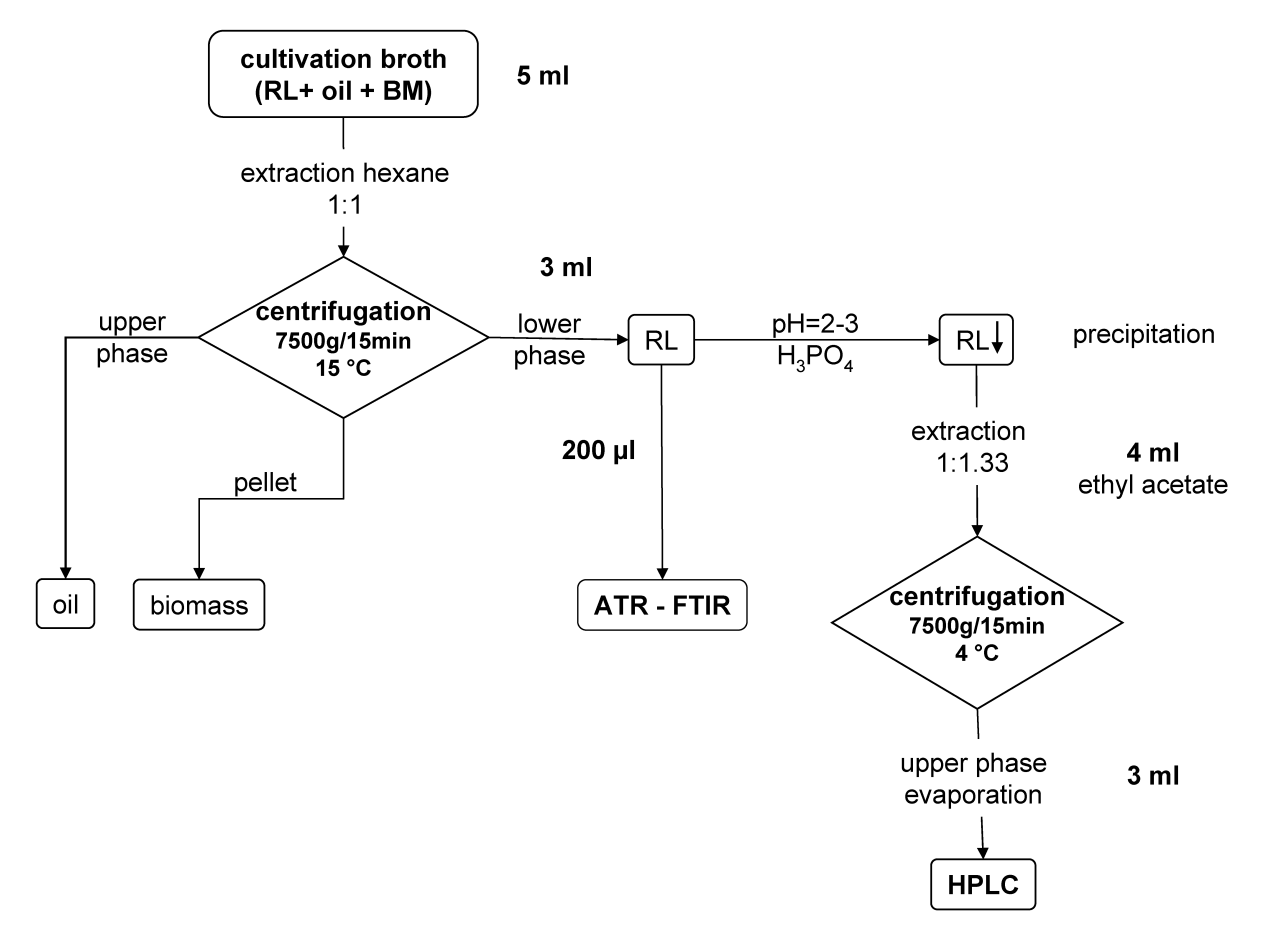
**Sampling procedures**. This figure shows the schematic proceedings for the applied sample extraction from culture broths.

The quantifications of bio dry weight, oil and rhamnolipid contents were performed according to the extraction protocol shown in Figure 4. For oil and cell separation, 5 ml of cultivation broth was mixed with 5 ml of hexane (to separate the nonpolar compounds) and centrifuged at 7500 g at 15°C for 15 minutes. 200 μl of the lower, aqueous phase was used for the ATR – FTIR analysis, as described below. For HPLC reference analysis, 3 ml of the lower phase were transferred and acidified with 85% H_3_PO_4 _to pH = 2 – 3. 4 ml of ethyl acetate were added for the extraction of the rhamnolipids. After mixing, the suspension was centrifuged at 7500 g at 4°C for 15 minutes. 2 × 1.5 ml of the upper phase were transferred to 2 ml cups and the ethyl acetate was evaporated at 60°C in a thermo mixer for 24 h and processed as described in the HPLC section.

### ATR-FTIR spectroscopy

**A**ttenuated **T**otal **R**eflectance (ATR) Fourier transform infrared spectra were collected with a bench-top spectrometer (Tensor 27, Bruker Optics GmbH, Ettlingen, Germany) equipped with a liquid nitrogen cooled, linear LN-MCT-Photovoltaic detector (Kolmar Technologies Inc., Newburyport, MA 01950, USA) and a BioATR II cell (Harrick Scientific Products Inc., Pleasantville, NY 10570, USA). The Bio-ATR II cell was purged by a continuous flow of dried air to minimize water vapour in the IR-beam path. The ATR cell was temperature stabilized at 25°C by a refrigerated circulator bath (HAAKE DC30-K20, Thermo Haake GmbH, Karlsruhe, Germany) to avoid water signals in the IR-sample spectrum resulting from temperature shifts between reference and sample measurement. Before the measurement, the crystal of the ATR cell was washed several times with 20 μl H_2_O. Cleanliness of the ATR-crystal was controlled by the measurement of a baseline of the water covered crystal before and after washing.

A reference spectrum of water was measured. Then the cell was washed twice with 20 μl sample and the actual sample measurement was performed. Both for reference and sample measurement, 64 interferograms were averaged resulting in an overall scanning time of about 1 minute. Spectra were recorded in a range from 850 – 4000 cm^-1 ^at a resolution of 4 cm^-1 ^and with an aperture of 6 mm. Data storage, spectra processing, substance comparison and the quantitative analysis of the spectra were done with the software OPUS 5.5 (Bruker Optics GmbH, Ettlingen, Germany).

### Data analysis

The recorded FT-IR spectra together with the results from HPLC reference analysis were analyzed using partial least-squares (PLS) regression. PLS calibration development, including cross- and test-set validation were performed with the QUANT 2 module of the OPUS 5.5 software, according to the multivariate calibration techniques described by Conzen [19]. Multiple mathematical pre-treatments were iteratively applied and for evaluation of the fits of the correlations to the reference HPLC data, the determination coefficients R^2 ^were calculated. Additionally, the predictive abilities of the correlation methods were proofed by calculation of two types of prediction errors. For validation within the whole calibration set, cross validations with one leave out sample were applied, and the root mean square errors of cross validation (RMSECV) were determined.

The evaluation of the predictive quality of an established calibration model applied on an independent test samples set from an arbitrarily selected fermentation process was done by test set validations and calculation of the root mean square error of prediction (RMSEP). All errors were calculated for different numbers of PLS factors. During the establishment of the calibration models, spectra were checked for outliners by visual inspection of principal component score plots. The final correlations were selected according to the lowest prediction errors and PLS ranks versus highest R^2 ^values were used to obtain confidential and stable calibration models.

### Reference rhamnolipid determination by HPLC

The ATR-FTIR analysis was calibrated and validated using HPLC as reference method. A rhamnolipid 1 standard was produced for the calibration of the HPLC device by enzymatic hydrolysis of rhamnolipid 3 [16] and purified by chromatography. At the stationary phase, fine silica gel was used, and at the mobile phase, a chloroform-methanol 75:25 was utilized. The rhamnolipid 3 standard with a purity grade of 97.3% was purchased from Hoechst AG (Frankfurt, Germany).

For UV-detection, the rhamnolipids were derivatized into esters of bromophenacylbromide. This was done using a 1:1 (v/v) derivatisation solution of 40 mM 4-bromophenacyle bromide and 20 mM triethylammonia in acetonitrile [20]. The rhamnolipid content of the samples was analyzed after redissolving the pellet in 1.5 ml of acetonitrile. A rhamnolipid content of approximately 0.1 – 1 mM was achieved by appropriate dilution. 1 ml of this solution was mixed with 200 μl of the derivatization agent. The derivatisation reaction took place at 60°C for 90 min. Subsequently, the rhamnolipids were separated in a reverse phase C_18 _column (Supelcosil LC-18, Supelco/Sigma-Aldrich cooperation Bellefonte, Pennsylvania, USA) on an HPLC device (Agilent 1100 series, Agilent Technologies GmbH, Germany) with a linear gradient of acetonitrile-water, and finally detected by a UV-detector at 265 nm. Solvent A consisted of 95:5 H_2_O/acetonitrile (v/v), solvent B was composed by 95:5 acetonitrile/H_2_O (v/v). Table 1 shows the applied HPLC gradient blending parameters., Retention times of 13.2 minutes and 15.17 minutes were determined for rhamnolipid 3 and rhamnolipid 1, respectively.

**Table 1:** Applied linear blending parameters for the solvents A & B for the HPLC rhamnolipid analysis.

**runtime**	**solvent A in %**	**solvent B in %**	**flow ml/min**
0.00	30	70	0.8
4.00	30	70	0.8
14.00	0	100	0.8
28.00	0	100	0.8
33.00	30	70	0.8

## Competing interests

The authors declare that they have no competing interests.

## Authors' contributions

FL, CS and RH coordinated the work in this project, gave intellectual courtesy and were involved in the writing work. FL performed the experiments and made the analysis presented in this publication.
